# Transient restructuring of the active oral resistome during probiotic *Streptococcus salivarius* K12 colonization in a 3D polymicrobial biofilm model

**DOI:** 10.1080/20002297.2026.2680793

**Published:** 2026-05-28

**Authors:** Nadeeka S. Udawatte, Chun Liu, Reuben Staples, Pingping Han, Purnima S. Kumar, Thiruma V. Arumugam, Sašo Ivanovski, Chaminda Jayampath Seneviratne

**Affiliations:** a The University of Queensland, School of Dentistry, Centre for Oral-facial Regeneration, Rehabilitation and Reconstruction (COR3), Brisbane, QLD, Australia; b Department of Periodontics and Oral Medicine, School of Dentistry, The Ohio State University, Columbus, OH, USA; c La Trobe Institute for Molecular Science, School of Agriculture, Biomedicine and Environment, La Trobe University, Melbourne, QLD, Australia

**Keywords:** *Streptococcus salivarius* K12, oral bacteria, probiotic colonization, active resistome modulation, metatranscriptomics, antibiotic resistance genes (ARGs), oral probiotic, oral 3D MEW mPCL polymicrobial biofilm model

## Abstract

**Background:**

The oral cavity harbours a complex and transcriptionally active antibiotic resistance gene (ARG) reservoir shaped by polymicrobial biofilm ecology. Whether probiotic-mediated ecological modulation can remodel the active resistome without promoting horizontal gene transfer remains poorly understood.

**Objective:**

To investigate the impact of *Streptococcus salivarius* K12 (Ssk12) colonisation on active resistome dynamics within saliva derived polymicrobial biofilms and determine whether probiotic driven ecological restructuring transiently alters resistance-associated transcriptional signatures.

**Design:**

Saliva-derived polymicrobial biofilms were established on three-dimensional melt electrowritten poly(ε-caprolactone) (MEW-mPCL) scaffolds and exposed to Ssk12. Metatranscriptomic profiling was performed across four time points (Baseline, Day 4, Day 7, and Day 10), complemented by quantitative PCR validation and ARG–mobile genetic element (MGE) co-localisation analysis to characterise resistome restructuring during probiotic colonisation and decolonisation.

**Results:**

Baseline biofilms contained 27 ARGs spanning 16 antibiotic classes, predominantly *ermB*, *tet(M)*, and *tet(W)*. During peak Ssk12 colonisation (Days 4–7), total ARG abundance declined to approximately 17% of baseline levels, with marked reductions in efflux-associated and β-lactam/fluoroquinolone resistance-associated transcripts. Partial resistome recovery occurred by Day 10 (~32% of baseline), indicating reversible ecological modulation rather than permanent dysbiotic restructuring. ARG dynamics were primarily reshaped by ARG-bearing taxa rather than enrichment of high-confidence putatively mobile resistance determinants.

**Conclusions:**

*S. salivarius* K12 transiently remodelled the transcriptionally active oral resistome within structured polymicrobial biofilms without evidence of enhanced putative horizontal resistance gene mobilisation. These findings support a proof-of-concept model in which probiotic driven ecological restructuring may create a transient resistome state potentially associated with altered responsiveness to selected antibiotic classes.

## Introduction

The human oral cavity harbours one of the most complex microbial ecosystems in the body, characterized by dense biofilms and rich taxonomic and functional diversity [1]. Among its microbial features, the oral resistome comprising antibiotic resistance genes (ARGs) embedded within commensal and opportunistic pathogens has emerged as a reservoir of concern, especially as horizontal gene transfer (HGT) events can occur in dense polymicrobial communities [2–4]. Recent metagenomic and systematic review-based analyses have demonstrated that saliva and supragingival biofilms contain diverse ARG repertoires enriched for resistance determinants targeting macrolides, tetracyclines, *β*-lactams and fluoroquinolones [5,6]. Among these, tetracycline resistance genes such as *tet(M)*, *tet(O)*, *tet(Q)* and *tet(W)*, along with ARGs associated with *Streptococcus* species, contribute substantially to the oral antimicrobial resistance landscape [7,8]. Emerging evidence demonstrates that environmental pressures such as chlorhexidine exposure can disrupt the oral microbiome and influence antibiotic resistance patterns, while oral streptococci may respond by altering their genetic and gene-expression profiles to survive antimicrobial stress [8]. Importantly, comprehensive investigations across clinical cohorts have detected dozens of ARGs spanning multiple antibiotic classes [9], while revealing a consistent discordance between genotypic detection and phenotypic resistance, underscoring that the mere presence of ARGs does not necessarily translate into functional expression [10]. Despite this diversity, the oral resistome remains relatively stable and resilient under healthy conditions, with a conserved ARG composition over time [11,12].

Probiotics are increasingly investigated as ecological modulators of microbial communities [13]; however, their effects on transcriptionally active ARGs within oral biofilms remain poorly defined. Experimental models suggest that probiotic exposure can alter ARG prevalence, yet distinguishing direct probiotic effects from broader microbiome restructuring remains challenging [14–16]. Changes in ARG abundance are often linked to shifts in bacterial community composition rather than the acquisition of new resistance determinants [17]. Genomic surveys have detected mobile resistance determinants, including *erm(B)*, *tet(M)* and *β*-lactamase genes, in some commercial probiotic strains [14], particularly in multi-strain formulations [18,19]. However, genomic detection alone does not establish transcriptional activity, phenotypic resistance, or horizontal dissemination within polymicrobial biofilms.

Despite increasing recognition of the oral cavity as a reservoir of antimicrobial resistance, most studies remain limited to DNA-based inventories of ARG carriage and therefore lack resolution regarding transcriptionally active resistome dynamics within structured oral biofilms [5,6,20]. Although many studies have provided valuable insights into probiotic detection at the genomic level and its association with ARG abundance [5], they often lack the resolution to capture active resistome dynamics to determine whether such activity originates from probiotic presence [9,21]. Despite the clinical relevance, it consequently remains unclear whether probiotic presence alters active ARG expression within polymicrobial oral communities or whether the observed changes primarily reflect ecological restructuring of the microbial community.

Probiotics such as *Streptococcus salivarius* K12 (Ssk12) have shown promise in reshaping microbial communities via niche competition [22], bacteriocin production [23], immune modulation [24,25], and, more recently, antiviral activity (24). A naturally occurring oral commensal, Ssk12 produces lantibiotics (e.g. salivaricins A2 and B) that inhibit Streptococcus pyogenes, *Staphylococcus aureus*, and other Gram-positive competitors [23]. Clinical studies report reduced frequency of upper respiratory tract infections following Ssk12 administration, supporting its role in maintaining oral and nasopharyngeal health [23,26,27]. Direct evidence linking probiotic Ssk12 to changes in transcriptionally active ARG expression within polymicrobial oral biofilms remains largely unexplored. Here, we characterize RNA-level resistome dynamics during Ssk12 colonization and following probiotic decolonization, with a particular focus on ARG–mobile genetic element associations as indicators of potential mobility rather than confirmed gene transfer.

Building on our previous work, we have shown that saliva-derived polymicrobial biofilms cultured in vitro on three-dimensional melt-electrowritten (MEW) medical-grade poly(*ε*-caprolactone) (mPCL) scaffolds accurately recapitulate the microbial composition, spatial organization, and proteo-transcriptomic functionality of naturally formed oral biofilms [28–31]. By mimicking key aspects of the oral microenvironment, this biomimetic system captures the molecular and ecological dynamics of polymicrobial communities within a controlled experimental setting [29]. Owing to its ability to recapitulate the structural and functional attributes of oral biofilms under controlled conditions, the MEW mPCL scaffold model serves as a versatile experimental framework for investigating biofilm-associated phenomena, including ARG dissemination associated with the modulatory effect of probiotic colonization within polymicrobial oral communities.

In the present study, we integrated a 3D salivary polymicrobial biofilm model with metatranscriptomic profiling to characterize temporal changes in the active oral resistome across different phases of probiotic SsK12 colonization. Specifically, we aimed to determine whether transient probiotic colonization is associated with community-level restructuring of resistome dynamics within a spatially complex polymicrobial biofilm ecosystem.

## Methodology

### Saliva collection and ex vivo 3D biofilm establishment

**Figure 1.**
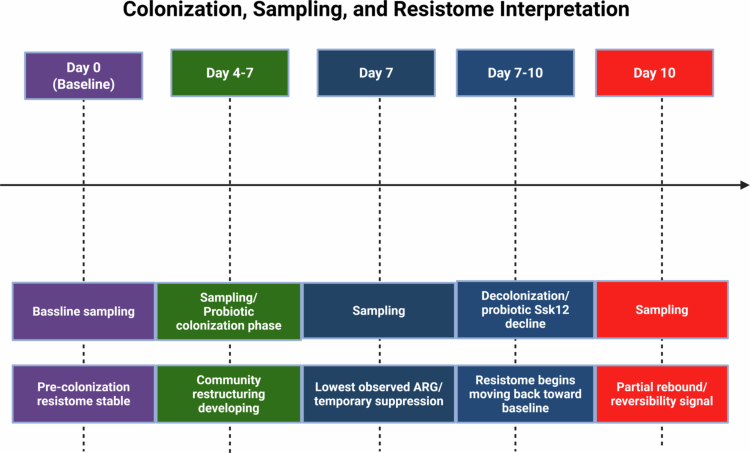
Schematic overview of probiotic Streptococcus salivarius K12 colonization, sampling timeline, and interpretation of resistome dynamics within the 3D polymicrobial oral biofilm model. Baseline (Day 0) represents the pre-colonization reference state, followed by transient probiotic colonization (Days 4–7), peak resistome restructuring at Day 4, and partial reversibility during probiotic decline/decolonization (Days 7–10). Sampling points were analyzed using RNA-based metatranscriptomic profiling to evaluate temporal changes in active ARG-associated transcriptional signatures.

Building on data from our previous study [29], unstimulated saliva was collected from four periodontally healthy adults (Supplementary Table S1) and used to establish 16 ex vivo biofilm samples cultured on 3D MEW mPCL scaffolds at four different time points (Baseline, Day 4, Day 7 and Day 10). Scaffolds were fabricated from medical-grade PCL (Corbion Inc., Australia, PURASORB PC 08) using melt electrowriting under optimized, controlled conditions, as described previously [28,29,31]. A schematic representation of the experimental design and 3D polymicrobial biofilm model is shown in Figure 1.

Briefly, three-dimensional fibrous scaffolds were fabricated from medical-grade poly(*ε*-caprolactone) (PCL; PURASORB PC 08, Corbion Inc., Australia) using melt electrowriting (MEW) under controlled processing conditions. Briefly, PCL was preheated to 85 °C for 1 h prior to printing and extruded through a syringe nozzle using 0.15 MPa air pressure. A voltage of 10 kV was applied between the nozzle and a movable aluminium collector positioned 8 mm apart, enabling precise deposition onto the collector at a translation speed of 1,060 mm/min. Scaffolds were fabricated with a defined architecture comprising ~250 μm pore size and 0.8 mm thickness, followed by punching into uniform 5 mm constructs. Custom deposition patterns were generated using G-code and controlled using Mach3 motion software (Artsoft, USA) developed by COR3 laboratory.

This scaffold platform was previously validated as a biomimetic 3D oral biofilm model, yielding thicker, more biomass-rich, and more viable polymicrobial communities that more closely reproduce the microbial complexity and compositional fidelity of naturally formed oral biofilms than conventional 2D culture systems [30]. Moreover, 16S rRNA gene sequencing of 4-day mature biofilms demonstrated that the 3D MEW-PCL scaffold system retained approximately 60% of the core oral microbiome at both the phylum and genus levels, indicating preservation of key microbial community structures within the biofilm model [30]. Biofilms were established in the absence of probiotic supplementation at baseline and subsequently sampled on Days 4, 7, and 10 following a single inoculation with *Streptococcus salivarius* K12 (Ssk12). These time points were selected to capture baseline conditions, transient probiotic colonisation (Days 4–7), and subsequent decolonisation (Day 10) within the model, as previously described [29]. The Ssk12 dosing was selected to achieve colonization levels consistent with those naturally observed in the oral cavity for this strain [32].

*Streptococcus salivarius subsp. salivarius strain K-12 [DSM 13084] obtained from ATCC (University Boulevard in Manassas, Virginia, USA)* was cultured using BHI broth (BD Biosciences) according to the manufacturer’s instructions provided (ATCC), cultured aerobically at 37 °C in an orbital shaker (150 rpm) for 18–24 h. Briefly, 1  mL of overnight culture (1 × 10⁷/mL) was inoculated into 10  mL brain heart infusion (BHI) broth and incubated for 24 h. Cultures were subsequently centrifuged at 5000 × g for 10 min, and the supernatant was sterile-filtered through a polyvinylidene fluoride membrane to generate spent culture medium (SCM) for downstream biofilm assays. Prior to inoculation, melt electrowritten poly(*ε*-caprolactone) (MEW-PCL) scaffolds were sterilized by immersion in 70% ethanol for 15  min followed by ultraviolet exposure for 20  min. Unstimulated saliva samples collected from four periodontally healthy donors were combined with defibrinated sheep blood and heart infusion medium at a ratio of 1:1:8 to establish polymicrobial biofilms. A total volume of 300  μL inoculum was added to 48-well plates containing scaffold and non-scaffold conditions and cultured for 10 days under anaerobic conditions (37 °C, 80 rpm) using a Whitley A20 anaerobic chamber (A20 Whitley Anaerobic Chamber, Bingley, West Yorkshire, UK) as described in the protocol established in our prior work [29].

Participant recruitment and study procedures were approved by the Human Research Ethics Committee of The University of Queensland (HREC No. 2023000467/06/2024). Eligibility followed the 2017 World Workshop on the Classification of Periodontal and Peri-Implant Diseases and Conditions, ensuring that participants had a PPD ≤ 3 mm, BOP ≤ 10%, and no history of periodontal disease. The cohort included four Asian individuals (2 males, 2 females; 37–40 years, mean 38.5 ± 1.3). The exclusion criteria included tobacco use, oral/systemic disease, recent orthodontic treatment (within 12 months), recent antibiotics, and recent use of prebiotics, probiotics, or antiseptic mouth rinses (within 3 months). The participants abstained from food and beverages for ≥1 h prior to collection. Saliva was preserved with 20% glycerol and stored at −80 °C until use.

### Longitudinal monitoring of SsK12 colonization in 3D salivary polymicrobial biofilms

Longitudinal colonization dynamics of Ssk12 within saliva-derived 3D MEW mPCL biofilms were previously characterised using RT-qPCR and smFISH imaging [28]. Briefly, a TaqMan assay targeting three regions of the *sbo* locus (*SboK*, *SboG* and *SboA*) confirmed Ssk12 establishment, while confocal smFISH imaging defined its spatial distribution within the biofilm matrix. These analyses demonstrated peak colonization at day 4, followed by a decline by Day 10 [29]. The same experimental cohort and biofilm samples were used here to investigate transcriptionally active ARG dynamics derived from mRNA sequencing.

### Metatranscriptomic sequencing (mRNA) and data processing of ex vivo 3D polymicrobial biofilms

Total RNA was extracted from ex vivo 3D polymicrobial biofilms as previously described [29], with minor modifications detailed below. The biofilm samples were enzymatically pretreated with lysozyme (5 mg/mL) in the presence of NaCl–EDTA activation buffer prior to mechanical disruption using 0.150–0.212 mm glass beads. Total RNA was subsequently extracted using the RNAzol® RT protocol following bead-assisted lysis and phase separation. The RNA was precipitated with isopropanol, washed with 75% ethanol, and resuspended in RNase-free water. RNA quality and quantity were assessed using NanoDrop spectrophotometry, Qubit fluorometric quantification, and Agilent 2100 Bioanalyzer analysis. Triplicate RNA extracts were pooled prior to library preparation to maximize RNA integrity and sequencing quality, yielding RNA integrity numbers (RIN) ranging from 6.8 to 8.0 and A260/280 ratios between 2.0 and 2.2 before downstream metatranscriptomic sequencing and analysis.

Strand-specific shotgun metatranscriptomic sequencing was performed to characterize community-wide transcription-associated signatures of the active oral resistome, whereas RT-qPCR was used as a targeted approach to monitor probiotic colonization and validate selected ARG-associated targets. Accordingly, the active resistome was operationally defined as ARG transcripts detected in RNA-derived libraries, which is distinct from a DNA-based inventory of genomic ARG carriage or resistance potential alone. The RNA-seq libraries were prepared using the Illumina Stranded Total RNA Prep Ligation Kit and Illumina RNA UD Indexes (Illumina, 20091655) according to the manufacturer’s protocol (Illumina, Document #1000000124514 v04), together with the Ribo-Zero Plus rRNA depletion Kit (Illumina, 20040529). This workflow selectively depletes abundant ribosomal RNA transcripts while preserving transcript strand orientation during library preparation, thereby enriching for non-ribosomal transcripts, including messenger RNA (mRNA). In addition, residual ribosomal RNA reads were computationally removed during downstream preprocessing using rRNA-filtering approaches (e.g. alignment against reference rRNA databases with tools such as SortMeRNA [33]), further enriching transcriptionally informative reads for resistome analysis. Consequently, transcript abundance was interpreted as evidence of transcription-associated activity at the microbial community level rather than as a direct surrogate of phenotypic resistance, and RNA-derived reads were not assumed to originate exclusively from mRNA under all conditions.

Briefly, Library preparation was performed from 80 ng of total RNA, which was depleted of abundant rRNA and then fragmented in a heat fragmentation step. cDNA was synthesized from the fragmented RNA using random primers. The first-strand cDNA was converted into dsDNA in the presence of dUTP to prevent subsequent amplification of the second strand and thus maintain the strand orientation of the original RNA. The 3’ ends of the cDNA were adenylated, and pre-index anchors were ligated. The libraries were then amplified with 14 cycles of PCR, incorporating unique indices for each sample to produce libraries ready for sequencing. The libraries were quantified on the Perkin Elmer LabChip GX Touch with the DNA High Sensitivity Reagent Kit (Perkin Elmer, CLS760672). Libraries were pooled in equimolar ratios. Sequencing was performed using the Illumina NextSeq500 (NextSeq control software v4.0.0/Real Time Analysis v2.11.3). The library pool was diluted and denatured according to the standard NextSeq protocol (Document # 15048776 v16) and sequenced to generate single-end 82 bp reads using a 150-cycle NextSeq500/550 High Output Reagent Kit v2.5 (Illumina, 20024907) (12–16 million reads/sample). Trimmed RNA-seq reads were assembled de novo using MEGAHIT v1.2.9 with iterative multi-k-mer assembly (k-mer range 21–141) to optimize transcript reconstruction and contig continuity [34]. Scaffolds longer than 500 bp were retained for downstream analyses, including gene prediction and statistical profiling. Processed reads were subsequently aligned to reference genomes using HISAT2 v2.2.1 for transcript quantification and resistome analysis [35].

### Identification of antibiotic resistance genes (ARGs) and mobile genetic elements (MGEs)

Antibiotic resistance genes (ARGs) were identified using Resistance Gene Identifier (RGI) against the Comprehensive Antibiotic Resistance Database (CARD v3.0.7) [36]. The CARD was selected for this study because of its expert-curated collection of experimentally validated resistance genes and resistance-conferring mutations integrated within the antibiotic resistance ontology (ARO), enabling the mechanistically interpretable classification of resistance determinants according to resistance mechanisms and antibiotic classes. The predicted open reading frames (ORFs) were translated and aligned to CARD reference sequences using curated BLASTP thresholds. Only hits classified as *Perfect* (100% reference coverage with ≥90% identity) or *Strict* (above curated bitscore cut-offs) were retained, and nudged hits were excluded to minimize false positives. Functional annotations, including resistance mechanisms, drug classes and ontology assignments, were retrieved by parsing CARD metadata files (card.json) following established workflows (https://card.mcmaster.ca/download) [36]. Genes were classified according to the Antibiotic Resistance Ontology (ARO), and resistance mechanisms were grouped into major functional categories, such as efflux transport, target modification, enzymatic inactivation and antibiotic degradation. As only CARD was used for ARG annotation, resistance determinants that are poorly characterized or underrepresented in this database, particularly those associated with non-culturable or non-clinical taxa, may not have been fully captured.

To assess potential ARG mobility, annotated genes were integrated with mobile genetic element predictions using MobileElementFinder (v1.0.3) (MGE) [37], and contig-level assemblies were screened for ARG–MGE co-occurrence to infer genomic linkage rather than confirmed horizontal transfer [38]. To evaluate the genomic context and potential mobility, the contigs were co-annotated for mobile genetic elements (MGEs) using the MobileOG database (v1.6) and MobileElementFinder (v1.0.3) with DIAMOND, applying ≥80% identity and fragment coverage thresholds. MGEs were classified as plasmids, phages, integrative genetic elements (IGEs), conjugative elements (CEs) or insertion sequences (IS).

Potential ARG co-localization was assessed by annotating contig-level assemblies for both ARGs and mobile genetic element (MGE) markers, enabling evaluation of the genomic context beyond sequence detection alone. ARG-carrying chromosomal contigs were assigned to bacterial taxa based on metagenome-assembled genome (MAG) classifications [39].

### Co-localization analysis and stochasticity analysis of co-localization

Co-localization refers to the physical proximity of genes located on the same assembly contig. To illustrate class 1 integron structures, representative plasmid-derived contigs were selected. As previously described [39], contigs with redundant gene combinations were deduplicated, and nested assemblies were removed to generate a representative set of co-localization contigs, following previously described approaches. Gene arrangements were visualised using the *gggenes* R package (https://wilkox.org/gggenes/) [40].

To evaluate the stochasticity of co-localization events, an enrichment score was computed as the ratio of the observed to expected number of co-localized contigs as previously described [39]:

$$\text{Enrichment Score}=\frac{N_{\text{observed}}}{N_{\text{expected}}}$$

where:

-
$N_{\text{observed}}$ = observed number of co-localized contigs
-
$N_{\text{expected}}$ = expected number of co-localized contigs under the null model

An enrichment score greater than 1 was interpreted as evidence of non-random enrichment within the salivary biofilm microbiome. The expected number of co-localization events was estimated as previously described [39]:

$$N_{\text{expected}}=N_{\mathrm{total}}\times p$$

where:

-
$N_{\text{expected}}$ = expected number of co-localised contigs
-
$N_{\text{total}}$ = total number of contigs
-
p = probability of co-occurrence under the null model

where *p* represents the expected probability of two genes co-occurring on the same contig. This was calculated as:

$$p=\left(\frac{N_{A}}{N_{\text{total}}}\right)\times \left(\frac{N_{B}}{N_{\text{total}}}\right)$$

where:

-
$N_{A}$ = number of contigs containing gene A
-
$N_{B}$ = number of contigs containing gene B

Statistical significance of ARG co-localization was assessed using a binomial framework with false discovery rate (FDR) correction implemented in R (*binom.test*). The number of observed co-localized contigs (*x*) was tested against the expected probability of co-occurrence (*p*) under a null model assuming random gene distribution across *n* total contigs. Adjusted *P*-values < 0.001 were considered indicative of non-random enrichment.

### RT-qPCR to validate expression profiling of the most dominant ARGs

Total RNA was extracted from biofilm samples collected at baseline and across subsequent Ssk12 colonization timepoints. To assess changes in transcriptional activity, core antibiotic resistance genes (ARGs) including *ermB*, *mgrA*, *TetM*, *tetW*, *saur_lmrS*, and *Bado_rpoB* that exhibited modulation greater than 13% were selected for quantitative reverse transcription PCR (RT-qPCR) analysis.

#### cDNA synthesis

Complementary DNA (cDNA) was synthesized from 500 ng of total RNA using the First Strand cDNA Synthesis Kit (ThermoFisher Scientific, Catalogue No: K1612), following the manufacturer’s instructions. No-reverse transcriptase (–RT) controls were included to confirm the absence of genomic DNA contamination.

#### Quantitative PCR (qPCR) for ARG expression

Quantitative PCR was performed using the SsoAdvanced™ Universal SYBR® Green Supermix (Bio-Rad) on a QuantStudio 6 Flex Real-Time System (Applied Biosystems). Each 20 µL reaction contained 10 µL SYBR Green Supermix, 0.4  µM of each primer, 2 µL of diluted cDNA (corresponding to 10 ng input RNA), and nuclease-free water. The cycling conditions were: 95 °C for 2 min (initial denaturation), followed by 40 cycles of 95 °C for 15 s and 60 °C for 30 s. Melt-curve analysis was conducted to verify product specificity.

All reactions were performed in technical triplicate. Expression levels of ARGs were normalized to the bacterial *16S rRNA* gene as a reference gene, and the relative expression was calculated using the 2^−ΔΔCt^ method.

#### Target genes primer sequences

Representative dominant antibiotic resistance genes (ARGs) exhibiting variation in response to SsK12 intervention were selected for RT-qPCR analysis. These included genes associated with multidrug resistance (*lmrS*), macrolide resistance (*ermB*), tetracycline resistance (*tetM*, *tetW*), *β*-lactam resistance (*mgrA*) and rifamycin resistance (*Bado_rpoB*), as identified from prior metagenomic studies/primer sequences and their established relevance in the oral microbiota. The primer sequences and amplification efficiencies are detailed in Supplementary Table S2.

### Statistical analysis

Relative ARG abundances were calculated as the mean gene coverage normalized to the total protein-coding gene coverage per metagenome. Data were transformed using a centred log-ratio (CLR) approach with a pseudocount of 1 × 10^−15^, and genes with zero abundance across all samples were removed. Differential abundance testing between the probiotic intervention groups and baseline controls was performed using ALDEx2 [41]. Owing to low variance and sparse ARG counts, standard DESeq2 dispersion modelling was not applicable [42]; therefore, gene-wise dispersion estimates were assigned directly as recommended for datasets with minimal dispersion heterogeneity. All analyses were conducted in R v4.5.0 (R Core Team, 2025) (https://www.R-project.org).

## Results

### Probiotic colonization is associated with a reduced ARG load in oral biofilms

**Figure 2.**
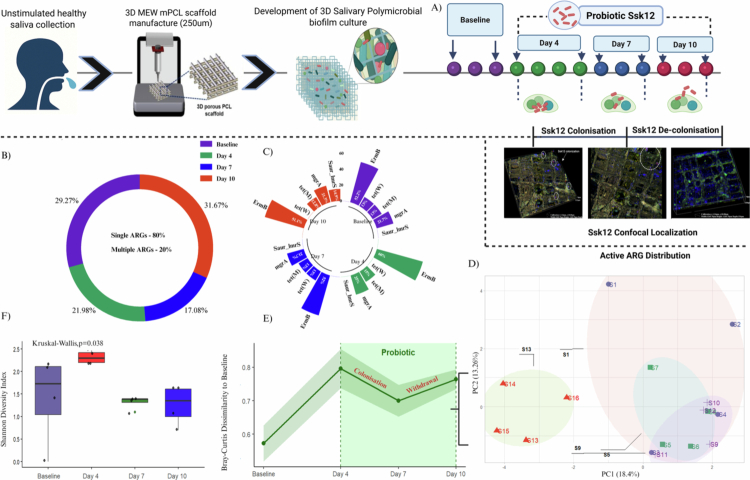
Experimental design and longitudinal modulation of the oral resistome during *Streptococcus salivarius* K12 colonization and withdrawal. (A) Schematic representation of the experimental workflow: unstimulated saliva was collected from healthy donors and inoculated into 3D melt-electrowritten (MEW) polycaprolactone (PCL) scaffolds (250  µm pores) to establish a 3D salivary polymicrobial biofilm model. The biofilm was treated with *Streptococcus salivarius* K12 (Ssk12) probiotic and sampled longitudinally at baseline, Day 4, Day 7 and Day 10, corresponding to the Ssk12 colonization and decolonization phases. Representative confocal images show Ssk12 localization within the biofilm matrix during colonization and its decline during decolonization, confirming spatial probiotic dynamics. (B) Principal coordinate analysis (PCoA) based on Bray–Curtis dissimilarity showing distinct clustering of samples across time points, reflecting compositional shifts in active ARG profiles during probiotic exposure and withdrawal. (C) Temporal dynamics of Bray–Curtis dissimilarity relative to baseline, demonstrating an initial divergence upon probiotic colonization (Days 4–7) followed by partial reversion during withdrawal (Day 10). (D) Changes in ARG diversity estimated by the Shannon index, with significant differences observed between time points (Kruskal–Wallis, *p *= 0.038). (E) Distribution of single- and multiple-resistance genes within the active ARG pool, highlighting the predominance of single ARGs (80%) and their temporal proportion across sampling points. (F) Circular plot illustrating temporal variation in key ARGs (*mgrA*, *tet*, *ErmB*, *Saur_lmrS*, *norA*) across time points, indicating transient shifts in relative ARG representation during colonisation and decolonization.

To assess the impact of probiotic Ssk12 on the oral biofilm resistome, we examined ARG profiles across three phases: precolonization (baseline), active colonization (Days 4 and 7), and post-colonization (Day 10) (Figure 2A). Principal coordinate analysis (PCoA) revealed a pronounced but reversible restructuring of the resistome during Ssk12 colonization. The baseline samples clustered tightly, whereas the Day 4 and Day 7 samples shifted markedly along PC1 (explaining 34.5% of variance), reflecting probiotic-driven changes; by Day 10, the profiles moved back toward the baseline centroid (ANOSIM *P *= 0.072) (Figure 2B). Boxplots of PC1 scores confirmed a rise during colonization and a decline after withdrawal. Bray–Curtis dissimilarity to baseline increased sharply at Day 4 (≈0.8) and remained elevated at Day 7, then partially decreased by Day 10 (≈0.75), indicating peak community restructuring during probiotic presence followed by partial recovery (Figure 2C). A Kruskal‒Wallis test revealed a significant difference in Shannon diversity among the experimental groups (*p *= 0.038) (Figure 2D), indicating that probiotic Ssk12 intervention influenced the overall diversity of ARGs within oral biofilms.

Collectively, these results demonstrate that Ssk12 colonization induces a clear, yet transient, shift in the oral resistome and is associated with a temporary reduction in ARG burden that diminishes once colonization subsides.

### Temporal reshaping of the active oral resistome during Ssk12 colonization

A total of 27 unique antibiotic resistance genes (ARGs) were identified across all study samples, encompassing 15 distinct resistance classes. Among these, 80% were classified as single ARGs, while 20% were multiple ARGs (Figure 2E). The highest ARG prevalence was observed on Day 10 (31.7%), followed by baseline samples without probiotic intervention (29.3%), Day 4 (22%), and the lowest on Day 7 (17.1%) (Figure 1F).

**Figure 3.**
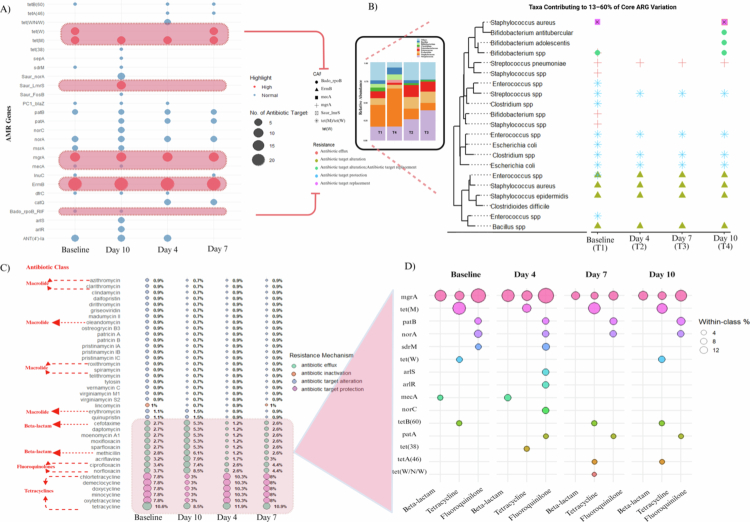
Functional classification of antibiotic resistance genes during probiotic Ssk12 intervention. (A) Differential abundance of ARGs annotated by CARD across time points. The bubble size denotes the number of antibiotic targets, and red shading indicates highly expressed genes. (B) Phylogenetic tree of taxa contributing to 13–60% of core ARG variation, showing the replacement of *Staphylococcus aureus*-associated ARGs at baseline by probiotic-influenced taxa during colonization, followed by re-emergence upon withdrawal. Stacked bar plot showing the total relative abundance of bacterial genera carrying active antibiotic resistance genes (ARGs) across experimental time points. The composition reveals temporal shifts in ARG-harbouring taxa during probiotic *S. salivarius* K12 colonization and withdrawal, with notable fluctuations in *Staphylococcus*, *Bifidobacterium*, *Streptococcus* and *Enterococcus* genera contributing to overall ARG burden dynamics. (C) Temporal shifts in antibiotic class–associated resistance profiles, illustrating shifts from a baseline predominance of macrolide- and *β*-lactam-associated resistance transcripts toward transient increases in tetracycline- and fluoroquinolone-associated signatures during probiotic colonization. (D) Within-class variation of key ARGs (*mgrA*, *tet(M)*, *patB*, *norA*, *sdrM*), showing dynamic modulation corresponding to *β*-lactam, tetracycline and fluoroquinolone classes.

Among the drug classes identified, more than half contained ARGs conferring resistance to multiple antibiotics. Notably, the most prevalent ARGs across the time points and baseline samples were ermB, mgrA, *tet(M)*, and *tet(W)*, with *ermB* consistently dominating at 50–60% across all groups. During Ssk12 colonization, notable shifts were observed in the composition of ARGs within the oral biofilm community. The tetracycline resistance genes *tet(W)* and *tet(M)* became particularly prominent, each accounting for approximately 13–15% of the total ARG abundance and associated with a high number of antibiotic targets (Figure 3A). In contrast, following Ssk12 decolonization, the multidrug efflux pump gene *lmrS* of *Staphylococcus aureus* emerged as a distinct marker, representing 14.9% of total ARGs. Additionally, Saur_norA/C, *lmrS*, *tet(38)*, and *sepA* were upregulated exclusively at Day 10, corresponding to the post-colonization phase. Conversely, genes such as *sdrM*, *mecA*, and *Bado_rpoB* exhibited downregulation during probiotic colonization (Days 4 and 7) but returned to baseline levels by Day 10 (Figure 3A).

Then, we investigated the contributing taxa responsible for 13–60% variation of core ARGs across the sampling points, focusing on *ermB*, *mecA*, *tet(M)*, *tet(W)*, *lmrS, mgrA* and *Bado_rpoB* (Figure 3B). A total of 11.3% of ARGs were associated with *streptococcal* species, an oral commensal species, followed by *Enterococcus*, *Esherichia coli* and *Clostridium* species (collectively 8.7%) at baseline. High-resolution mapping of ARGs to their contributing ARG-carrying species revealed pronounced temporal shifts with Ssk12 colonization (Figure 3B). At baseline (T1), ARGs were confined to a limited set of commensals, primarily *Enterococcus* spp. and *Bifidobacterium* spp., carrying tetracycline resistance genes [*tet(M)*, *tet(W)*] and a minority of efflux determinants. By Day 4 (T2), coinciding with peak Ssk12 colonization, ARG diversity and abundance expanded markedly, with *ermB* and *β*-lactam target-alteration genes (*mgrA*, *lmrS*) detected across multiple Firmicutes (*Staphylococcus aureus, Bacillus spp., Enterococcus spp*.) and tetracycline genes spreading to *Escherichia coli* and *Clostridium* spp. During continued colonization (T3), ARG profiles remained elevated but began to contract, retaining mainly *ermB* and *Tet* variants, which is indicative of a transient yet partially stabilized resistome. By Day 10 (T4), following Ssk12 decline, the ARG landscape largely reverted toward baseline, with most *mecA* and *ermB* signals lost and tetracycline determinants reduced, while a subset, including *Bado_rpoB*, persisted in select *Bifidobacterium* sp. Differential abundance analysis was performed using ALDEx2, which was preferred over DESeq2 because of the sparse and low-variance nature of the compositional ARG dataset. However, no significant shifts in ARG profiles or ARG-associated taxa were detected following SsK12 intervention.

### Probiotic *S. salivarius* K12 transiently reshapes the oral resistome

Next, we evaluated the antibiotic classes associated with the ARGs that were most influential under the influence of Ssk12 colonization with respect to both suppression and susceptibility patterns. ARGs were detected against 16 antibiotic classes, with the highest proportions observed for tetracyclines (8–12%), fluoroquinolones (2–6%), *β*-lactams (1–6%) and macrolides (1–2%) in the baseline biofilm (Figure 3C). Tetracycline resistance increased transiently (>10%) during Ssk12 colonization and subsequently decreased below baseline levels (3–8%) after probiotic decolonization. In contrast, resistance to acriflavine (7.9%) and the fluoroquinolones, ciprofloxacin (7.4%) and norfloxacin (8.5%) increased markedly during Ssk12 decolonization compared with both baseline (2–3.5%) and probiotic-colonized states (<3%) (Figure 3C). The temporal presence of SsK12 influenced resistance mechanisms in a time-dependent manner. At Days 4 and 7, efflux-associated pathways were markedly reduced, while target protection mechanisms mainly conferring tetracycline resistance were proportionally increased. By Day 10, following probiotic decolonization, efflux mechanisms became dominant (52.1%), and resistance levels exceeded those observed at baseline (Figure 3C).

The drug classes most impacted by Probiotic Ssk12 colonization were *β*-lactams, fluoroquinolones, and tetracyclines (Figure 3C). Consequently, we examined the specific ARGs exhibiting the greatest variation that could influence both susceptibility and suppression of these drug classes (Figure 3D). Among these, *mgrA* emerged as the most prominent ARG, showing variations of 6.2–15.7% for fluoroquinolones, 3.1–7.8% for *β*-lactams, and 3.1–3.9% for tetracyclines across different colonization phases. This was followed by *tet(M)*, which accounted for 2.1–8.7% of the tetracycline-associated variations from colonization to decolonization. All other ARGs contributed less than 2% to the observed changes in resistance for these drug classes.

Our data indicate that peak colonization of the probiotic Ssk12 at Day 4 was associated with a selective modulation of antibiotic resistance within the healthy, antibiotic-naïve oral biofilm. During this period, resistance to *β*-lactam and fluoroquinolone antibiotics transiently decreased by approximately 44% relative to baseline, suggesting enhanced susceptibility to these drug classes, whereas tetracycline resistance transiently increased by approximately 75.7%, indicating reduced tetracycline efficacy. Following the decolonization of the oral biofilm, these effects were partially reversed: resistance to tetracyclines declined, rendering the community more susceptible to tetracycline therapy, while resistance to *β*-lactams and fluoroquinolones returned toward baseline levels. Notably, variations in the core ARGs, particularly *mgrA* and *tet* genes, were the most influential genes contributing to these shifts in the antibiotic response window influenced by Ssk12.

### Transient resistome shifts without enrichment of mobilizable determinants during Ssk12 colonization

Given the transient resistome shifts observed during *Ssk12* colonization, we next examined whether probiotic Ssk12 colonization was associated with changes in ARG–mobile genetic element (MGE) co-localization within the oral biofilm.

To evaluate putative mobility-associated resistome features, assembled contigs were co-annotated for antibiotic resistance genes (ARGs) and mobile genetic elements (MGEs), and physical linkage was defined as ARG–MGE proximity on the same contig. Across all time points, 66 unique ARGs and 41 unique MGEs yielded 20 ARG–MGE co-localization pairs. No pairs met the conservative ≤10 kb proximity threshold used to define high-probability mobilizable resistance determinants (Supplementary Figure S1A, B). This cutoff was selected based on the universal 10  kb separation criterion applied by Kaszab et al. [43], who considered this threshold conservative relative to those used by other investigators [37,44,45]. Accordingly, no detectable high-confidence putatively mobile ARGs were identified under the applied criteria.

This pattern persisted throughout Ssk12 colonization, with no plasmid- or phage-associated ARGs detected within 10 kb at any time point (Supplementary Figure S1C). Enrichment modelling showed no deviation from random expectation, suggesting that the observed co-occurrence reflected incidental genomic proximity rather than biologically meaningful enrichment. Although total ARG–MGE co-localized contigs (>10 kb) varied modestly over time, these events occurred beyond conservative mobility thresholds and therefore do not support the expansion of a high-probability mobilizable resistome potential.

Contig-level co-localization analysis further supported this interpretation. ARGs were sparsely distributed across assemblies, and most contigs lacked detectable ARG–MGE linkage. Observed co-localization frequencies were at or below random expectation (Supplementary Figure S1D). After correction for multiple testing, no ARG pair showed significant enrichment relative to baseline (FDR q > 0.05), indicating limited evidence for MGE-mediated dissemination following probiotic Ssk12 colonization.

Collectively, these findings indicate that SsK12 colonization was not associated with detectable enrichment of high-confidence ARG–MGE linkage signatures within the salivary polymicrobial biofilm under the conditions tested. Although minor temporal fluctuations in ARG–MGE associations were observed, suggesting that transient resistome changes were more likely driven by community restructuring than by the enrichment of putatively mobile resistance determinants.

### Re-validation of core ARGs through RT-qPCR assessment

ARGs showing core modulation of ~13–60% across the oral biofilm resistome (Supplementary Figure S2) in all the groups, including *ermB*, *mgrA*, *tet(M)*, *tet(W)*, *lmrS* and *Bado_rpoB*, were selected for RT–qPCR validation following Ssk12 colonization. At baseline, transcriptional levels were low across all genes (Supplementary Figure S2). By Day 4, *ermB* and *mgrA* increased markedly and reached peak expression at Days 7 and 10, which was consistent with the metatranscriptomic trends. *lmrS* and *Bado_rpoB* showed delayed induction, with higher expression detected only at Day 10 during the post-colonization phase, which is also aligning with the metatranscriptomic data. In contrast, qPCR analysis revealed persistently low abundance of *tet(M)* and *tet(W)* across all time points, differing from the expression patterns observed in the metatranscriptomic data. Taken together, the RT–qPCR data provided limited but supportive evidence for the metatranscriptomic observations, indicating a reversible, time-dependent shift in ARG activity during *S. salivarius* K12 intervention.

## Discussion

The present study demonstrated that probiotics *Streptococcus salivarius* K12 are able to transiently remodel the oral resistome, shifting antimicrobial resistance patterns in a targeted manner without detectable enrichment of putative high-confidence ARG–mobile genetic element co-localization signatures. During Ssk12 colonization, the resistome shifted toward reduced expression of *β*-lactams and fluoroquinolones-associated ARGs, alongside a transient increase in tetracycline-associated ARGs. Given the re-emergence of the resistome by decolonization, we interpret the observed pattern as a temporary suppression or transient reshaping of the active resistome during peak probiotic colonization, followed by a partial rebound toward baseline as colonization declined, rather than as a sustained reduction.

The ARG profiles derived from our in vitro 3D salivary biofilm model represent the transcriptionally active resistome, as they are based on RNA-level data (meta-transcriptomics) rather than genomic presence alone. This approach captures ARGs that are actively expressed within the polymicrobial biofilm microenvironment and therefore may better reflect functional resistance potential under specific ecological conditions. Importantly, this addresses a key limitation in previous oral resistome studies, where shotgun metagenomics and phenotypic susceptibility testing frequently yielded discordant resistance profiles, underscoring the difficulty of inferring functional AMR solely from genomic presence [9]. Within oral biofilms, the dense and spatially structured microbial architecture further promotes close microbial interactions and may facilitate the exchange of ARGs carried on mobile genetic elements [46,47]. Consequently, the oral cavity has emerged as a clinically relevant reservoir of antimicrobial resistance, particularly given evidence that oral bacteria can disseminate to distal body sites and contribute to the gut resistome to a greater extent than previously recognized [48]. In this context and given the widespread and frequently unnecessary prescription of antibiotics in dental practice, the implementation of prudent antimicrobial stewardship strategies remains strongly recommended in clinical settings [9,49,50]. Compared with conventional metagenomics, the meta-transcriptomic approach used in our study enabled the detection of dynamic, condition-dependent shifts in ARG expression across baseline, Ssk12 colonization, and post-decolonization phases, providing ecological insight into active resistome modulation within polymicrobial oral biofilms. Although phenotypic resistance was not directly assessed, the healthy salivary-derived 3D oral biofilm model offered a rigorously controlled platform to investigate AMR ecology, microbial persistence, and community restructuring while minimizing host-related confounders inherent to clinical studies. Moreover, the 3D biofilm architecture recapitulated key structural and ecological features of native oral microbial communities, supporting the biological relevance of the observed transcriptional patterns. Nevertheless, these findings should be interpreted as a proof-of-concept mechanistic ecology study rather than direct evidence of clinical efficacy or AMR mitigation.

The human oral cavity harbours a rich and diverse resistome. Systematic surveys report on the order of 150–160 distinct ARGs in six oral sites (supragingival/subgingival plaque, mucosa, saliva, root canals, oropharynx) spanning ~22 antibiotic classes [9], contributing between 18% (mucosa) and 90% (subgingival biofilm) of the oral resistome. Among the predominant determinants, tetracycline resistance genes are the most widespread, representing up to 18–90% of ARGs in different oral sites. Although the detection of specific antibiotic resistance genes does not necessarily confirm phenotypic resistance in the specimen, it reflects the genetic potential for the expression of the corresponding resistance proteins [21]. Core tetracycline resistance genes such as *tet(M*) and *tet(O)* (ribosomal protection) and *ermB* (macrolide methylase) are consistently detected across oral niches [9]. *β*-lactam and macrolide–lincosamide resistance genes are also prevalent, likely reflecting the frequent clinical use of penicillins, clindamycin, and erythromycin in dental practice [51]. It indicates the potential for the expression of the encoded protein. Consistent with these reports, our baseline oral biofilm resistomes (27 ARGs across 16 classes) were dominated by *ermB*, *tetM*, *tetW*, and related determinants. Tetracycline (8–12%), fluoroquinolone (2–6%), *β*-lactam (1–6%) and macrolide (1–2%) resistance genes in the baseline samples closely aligned with previous findings. Most individuals harboured a single detectable ARG (~80%), which is consistent with the typical commensal oral microbiota, where individual species carry few resistance genes. Deep sequencing analyses have similarly reported dozens of ARGs (mean ~34) and multiple ARG-bearing taxa per individual [9]. Notably, *streptococcal* [9] commensals account for a major fraction (~20%) of the oral resistome (in our study, 11.3% of ARGs were associated with *Streptococcus* species, followed by *Enterococcus*, *Escherichia coli*, and *Clostridium* species (8.7%) at baseline. Collectively, our findings reaffirm that the healthy oral microbiome harbours a complex yet relatively stable resistome characterized by a consistent signature of tetracycline and macrolide resistance genes [9].

### Ssk12 induces a reversible shift in resistance signatures, with reduced efflux-associated ARGs and increased tetracycline-associated determinants during colonization

Introduction of Ssk12 altered the baseline resistome profile in the 3D polymicrobial oral biofilm model (Figure 1A–C). During active colonization (Days 4–7), the overall ARG expression signal decreased by 17% Compared to the baseline. Moreover, the resistome profile showed tighter PCA clustering reflecting reduced ARG diversity and homogenized resistance patterns. These findings are consistent with prior studies demonstrating that probiotics can reduce the ARG burden [15–17]. Notably, the resistome profile rebounds following the probiotic decolonization in the oral biofilm.

At the community level, Ssk12 colonization was associated with the shift in oral resistome from broad-spectrum efflux-mediated resistance toward ribosomal protection-type mechanisms. Tetracycline resistance genes (*tetM*, *tetW*) became proportionally more prominent (13–15%), while multidrug efflux determinants, including *norA/C*, *tet(38)*, *sepA*, *sdrM*, *mgrA* and *mecA*, showed a marked reduction in relative abundance. This redistribution indicates a restructuring of the resistome composition during SsK12 colonization, with decreased representation of efflux-associated resistance and a relative enrichment of ribosomal protection-associated transcripts. However, given the community-based nature of the metatranscriptomic data, these shifts likely reflect combined effects of changes in taxonomic composition and transcriptional activity, rather than direct selective suppression of specific resistance mechanisms.

Functionally, Ssk12 colonization was associated with an approximate 44% reduction in the relative abundance of transcripts linked to *β*-lactam and fluoroquinolone resistance, indicating a shift in the resistome composition during probiotic colonization. In contrast, tetracycline resistance-associated transcripts showed a transient increase during colonisation, which is consistent with elevated expression of *tet(M)* and *tet(W)* ribosomal protection genes. Notably, these tetracycline-associated signatures declined following probiotic decolonization, returning towards baseline levels. Overall, Ssk12 colonisation was associated with a reduction in the relative abundance of efflux-linked resistance transcripts while temporarily redistributing resistance-associated expression profiles, suggesting selective reorganisation rather than uniform suppression of resistance traits.

Ecologically, these shifts reflect the adaptive capacity of Ssk12, a commensal species equipped with potent bacteriocins and salivabactin that reprogram competitive hierarchies in the oral niche [23]. Ssk12 harbours a 190  kb plasmid encoding salivaricins A2/B and a newly identified salivabactin with activity against *Streptococcus pyogenes* (GAS), in an environment that is conducive to its antimicrobial activity. Such an inhibitory trait could suppress specific Gram-positive species competitors, such as *Streptococcus* and *Staphylococcus,* that typically contribute to the multidrug efflux systems within the oral biofilms [52]. By altering competitive hierarchies and niche occupancy, Ssk12 may indirectly reduce the abundance of taxa expressing multidrug efflux-associated ARGs under these conditions. Variations in the core ARGs, particularly *mgrA* and *tet* genes, were the principal determinants driving these observed shifts in this antibiotic response window. Among these, *mgrA*-associated lineages, predominantly *Staphylococcus* species, showed increased prominence relative to baseline, coinciding with a reduction of taxa that were initially dominant, which is consistent with our previous study [29].

### Resistome rebound following Ssk12 decolonization in oral biofilms

The probiotic-induced resistome shift was transient and reversible. By day 10, following Ssk12 decolonization, overall ARG expression increased towards baseline levels, reaching approximately 32% of baseline abundance. Resistance signatures associated with efflux-mediated mechanisms re-emerged as dominant (~52% of ARGs). Notably, *Staphylococcus aureus lmrS*, a multidrug efflux pump conferring resistance to fluoroquinolones and biocides, became a prominent marker, accounting for ~14.9% of post-withdrawal ARG expressions (Figure 2B). Other efflux-associated markers linked to acriflavine and fluoroquinolone resistance also reappeared (Figure 2C), indicating opportunistic recolonization by efflux-rich taxa once Ssk12 vacated its ecological niche.

This rebound pattern is consistent with the microbial resilience dynamics, in which transient ecological perturbations, whether probiotic or antibiotic, temporarily compress community diversity and resistance expression, followed by recovery toward a new equilibrium after the selective pressure ceases is removed. Similar dynamics have been reported in the gut microbiome [17], where probiotic interventions transiently suppress ARG expression, but allow re-expansion of resistance-associated taxa following withdrawal. Extending this paradigm to the oral cavity, our findings indicate that SsK12-driven resistome modulation is time-limited in the absence of sustained probiotic colonization.

Following Ssk12 decolorization, *Bifidobacterium* abundance increased, accompanied by the reappearance of the *rpoB* rifampicin-resistance marker (Figure 2A). The observed change reflects the intrinsic genetic background of these bacteria that recolonized the biofilm, rather than active selection or gene transfer driven by the probiotic Ssk12. This observation is consistent with our previous findings [29], which showed that *Bifidobacterium* levels significantly increase by day 10 during Ssk12 decolonization.

Collectively, these data indicate that the oral resistome not only rebounds but also reconfigures toward efflux-dominated resistance signatures, suggesting that sustained or repeated probiotic exposure may be required to maintain a resistome profile with reduced multidrug resistance potential.

### Ssk12 colonisation is not associated with enrichment of putative ARG–MGE linkage in oral biofilms

Previous studies of clinical and commensal *Streptococcus salivarius*, primarily based on qPCR, MLST typing, and antimicrobial susceptibility testing, have reported [53] the presence of resistance genes such as *tet(M)*, *erm(B)*, and *mef(A/E)*, which are occasionally associated with mobile genetic assemblies such as the MEGA element. While these isolate-level analyses have provided valuable insights into the intrinsic resistome of individual *S. salivarius* strains, they have offered limited resolution regarding active gene mobility or ecological behaviour within complex polymicrobial biofilms. In contrast, our study employed a polymicrobial oral biofilm model coupled with transcriptome-level resistome profiling, enabling direct evaluation of active ARG expression and potential mobilization in situ during different phases of probiotic colonization. However, it is important to note that this approach does not directly measure horizontal gene transfer (HGT) or ARG mobility.

Notably, no ARG–MGE pairs met the conservative ≤10 kb proximity threshold commonly used to define high-probability mobilisable resistance transcripts at baseline or during probiotic colonization (Supplementary Figure S1). Furthermore, no plasmid- or phage-associated ARGs were detected within 10 kb at any time point, and enrichment modelling demonstrated no statistically significant deviation from random co-occurrence. Although modest temporal fluctuations in ARG–MGE co-localized contigs (>10 kb) were observed, including a transient increase during Day 7 colonization, these events occurred beyond conservative mobility thresholds. Importantly, the absence of high-confidence mobilisable ARGs at baseline indicates that the healthy salivary polymicrobial biofilm in this model is enriched for putatively mobile resistance determinants. Although the few ARG–MGE co-localization events detected were phage-associated, their low frequency and lack of statistical enrichment do not support preferential phage-mediated mobilization.

Mobile genetic elements (MGEs) frequently harbour antibiotic resistance genes (ARGs) and virulence factors [12]. A decline in MGE-associated genomic contexts suggests a reduced potential for horizontal gene transfer, which may translate clinically into a lower risk of disseminating resistance determinants or pathogenic traits within the oral microbiome [54]. However, as HGT processes were not directly measured in this study, the observed lack of ARG–MGE enrichment should not be interpreted as evidence of reduced transfer potential. Instead, these findings indicate that Ssk12 colonization was not associated with detectable changes in the co-localization patterns of ARGs and MGEs under the conditions tested. Collectively, these data provide ecological insight into active resistome organization within a polymicrobial oral biofilm system and extend the current understanding beyond conventional isolate- or DNA-based analyses. While Ssk12 colonization was not associated with increased ARG–MGE linkage signatures, the transfer potential of resistance determinants within oral biofilms remains unresolved and will require targeted functional investigation in future studies.

While overall ARG expression was reduced during Ssk12 colonization, resistance transcripts were redistributed in parallel with taxonomic shifts rather than permanently eliminated or horizontally disseminated. These findings are consistent with the established safety profile of Ssk12 and related strains, which harbour only intrinsic resistances (e.g. modest aminoglycoside and fluoroquinolone tolerance), but not acquired ARGs [53]. This ecological modulation aligns with the established safety profile of Ssk12 and related strains, which harbour only intrinsic resistance traits and produce bacteriocins (salivaricins) that suppress susceptible taxa [52].

However, as shown in the gut microbiome studies [17], probiotic-induced perturbations can sometimes facilitate the expansion of non-probiotic resistances. In line with this paradigm, our findings further warrant that Ssk12 primarily restructure the oral resistome indirectly through shifts in the abundance of taxa already harbouring higher ARG burdens, rather than through the acquisition of new resistance genes.

Despite providing insights into Ssk12-mediated modulation of the active oral resistome, several limitations of this study should be acknowledged. First, the study employed an in vitro 3D salivary biofilm model derived from only four periodontally healthy donors, which may not capture the full inter-individual variability or complexity of the in vivo oral microbiome, particularly in disease contexts. Second, the temporary suppression was limited to ten days and single probiotic administation, constraining assessment of long-term or repeated probiotic interventions. Third, although ARG expression was quantified using metatranscriptomics and core resistance genes were validated by qPCR, we did not perform functional assays to confirm whether these genes confer phenotypic antibiotic resistance. As such, the observed changes in transcript abundance cannot be directly interpreted as functional antibiotic resistance, and phenotypic validation (e.g. MIC testing, antibiotic challenge, or biofilm survival assays) is required in future studies. In addition, our study did not directly assess ARG mobility, plasmid or integron dynamics, donor‒recipient exchange, or other transfer events. Consequently, our findings should not be interpreted as evidence that probiotic colonization decreased horizontal gene transfer potential. Rather, the data support transient ecological restructuring of community-level ARG expression within a polymicrobial oral biofilm. The relevance of HGT to this system remains an important question for future work using transfer-resolved and mobile-element-resolved approaches. Finally, while the healthy saliva-derived 3D oral biofilm model enabled controlled investigation of probiotic colonization and resistome dynamics, it does not fully recapitulate in vivo oral conditions, including host immunity, salivary flow, dietary exposure, and oral hygiene behaviours. Consequently, the translational relevance of these findings requires further validation in longitudinal and clinically representative studies.

## Conclusion

Our new findings contribute to the growing understanding of context-dependent probiotic–resistome interactions in structured poly-microbial communities. *S. salivarius* K12 transiently modulated the active oral resistome by suppressing multidrug-resistant taxa. Ssk12 colonization suppressed multidrug-resistant taxa, notably reducing efflux-pump genes while favouring ribosomal-protection (*Tet*) genes, leading to decreased ARG diversity and community convergence. These effects were reversible following probiotic decolonization, reflecting reversible, colonization-dependent dynamics. Hence, the limited intrinsic resistome associated with Ssk12 was not linked to detectable enrichment of putative ARG–mobile genetic element. Together, these findings suggest that the oral probiotic SsK12 was associated with transient restructuring of resistance-associated signatures within structured oral biofilms, while the broader resistome architecture remained comparatively resilient and was influenced by community-level ecological dynamics rather than direct probiotic effects alone.

## Supplementary Material

Supplementary Material Supplimentary Document.pdf

## Data Availability

Raw metatranscriptomic sequencing reads generated in this study have been submitted to the NCBI Sequence Read Archive (SRA) under submission ID SUB16135300 and will be publicly available upon assignment of a BioProject accession number. All other data supporting the findings of this study are available from the corresponding author upon reasonable request.
