# Supplementary material for: Transient restructuring of the active oral resistome during probiotic Streptococcus salivarius K12 colonization in a 3D polymicrobial biofilm model
Source: J Oral Microbiol. 2026 May 28;18(1):2680793. doi: 10.1080/20002297.2026.2680793 (PMC13224720; doi:10.1080/20002297.2026.2680793)
Supplement: Supplementary Material — Supplimentary Document.pdf [file ZJOM_A_2680793_SM8350.pdf]

Transient Restructuring of the Active Oral Resistome During *Streptococcus salivarius* K12 Colonization in a 3D Polymicrobial Biofilm Model

Nadeeka S. Udawatte[1], Chun Liu[1], Reuben Staples[1], Pingping Han [1], Purnima S. Kumar [2], Thiruma V. Arumugam[3], Sašo Ivanovski[1], Chaminda Jayampath Seneviratne [1,*]

1. The University of Queensland, School of Dentistry, Center for Oral-facial Regeneration, Rehabilitation and Reconstruction (COR3), Brisbane, QLD 4006, Australia.
2. Department of Periodontics and Oral Medicine, School of Dentistry, The Ohio State University, Columbus, OH 43210, USA.
3. La Trobe Institute for Molecular Science, School of Agriculture, Biomedicine and Environment, La Trobe University, Melbourne, QLD 3086, Australia.

Corresponding authors:

S.I.: s.ivanovski@uq.edu.au

J.S.: jaya.seneviratne@uq.edu.au

Table S1. Demographic Characteristics of Study Participants

	Participants (n=4)	
**Gender**	Male	2 (50%)
	Female	2 (50%)
**Age**	38.5 ± 1.29 (37, 40)	
**BOP**	1.8% ± 0.54 (1%, 2.2%)	
**PI**	0.075% ± 0.07 (0%, 0.18%)	
**Ethnicity**	Asian (100%)	

Data are displayed as mean ± standard deviations, n=4. BOP: bleeding on probing; PI: plaque index.

Table S2. Target Genes Primer Sequences of ARGs for RT-qPCR analysis

Target genes (ARGs)	Resistance class	Forward primer (5′–3′)	Reverse primer (5′–3′)	Annealing T a (°C)	Amplicon size (bp)	Reference / source
**ermB**	Macrolide–lincosamide–streptogramin B methyltransferase	GAAAAGGTACTCAACCAAATA	AGTAACGGTACTTAAATTGTTTAC	50	466-639	*Roberts MC,1999 (1)*
**tet(M)**	Tetracycline ribosomal protection	GTGGACAAAGGTACAACGAG	CGGTAAAGTTCGTCACACAC	55	406	*Ng LK,2001 (2)*
**tet(W)**	Tetracycline ribosomal protection	GAGAGCCTGCTATATGCCAGC	GGGCGTATCCACAATGTTAAC	64	168	*Ng LK,2001 (3)*
**mgrA**	Multidrug regulator / efflux-associated	GGGATGAATCTCCTGTAAACG	TTGATCGACTTCGGAACG	50	131	*Santos Costa S,2015 (3)*
**lmrS**	Multidrug efflux pump	TAACAACCCGGAGACGAAAC	CCCATCGCCGCTAAGTATA	58	241	*Mohammed,2020 (4)*
**Bado_rpoB**	Rifamycin target-site marker	GTGTGGTCCGCGAACGTATGA	AGGATGACGTCGCCGGAAT	60	309	*Lokesh D,2018 (5)*

Table S3. ARG–MGE co-localization within 150 kb in saliva-derived polymicrobial biofilms during different phases of Ssk12 colonization

Group	Ssk12 Colonisation Phase	ARG (≥90% identity)	ARG_ORF	MGE_Type	MGE_Category	Within 100-150kb
**Baseline**	-	patB	k95_8075	Phage	replication,infection,regulation	TRUE
**Baseline**	-	patA	k95_8075	Phage	replication,infection,regulation	TRUE
**Day4**	Colonisation	PC1 beta-lactamase (blaZ)	k95_2137	Other_MGE	competence	TRUE
**Day4**	Colonisation	PC1 beta-lactamase (blaZ)	k95_2137	Other_MGE	regulation	TRUE
**Day4**	Colonisation	mgrA	k95_2808	IGE	replication/recombination/repair	TRUE
**Day4**	Colonisation	mgrA	k95_2808	IGE	replication/recombination/repair	TRUE
**Day7**	Colonisation	mgrA	k95_2808	IGE	replication,inversion	TRUE
**Day7**	Colonisation	mgrA	k95_2808	IGE	replication,inversion	TRUE
**Day7**	Colonisation	msrA	k95_2916	Other_MGE	chaperone	TRUE
**Day7**	Colonisation	msrA	k95_2916	Other_MGE	chaperone	TRUE
**Day7**	Colonisation	msrA	k95_2916	Other_MGE	chaperone	TRUE
**Day7**	Colonisation	msrA	k95_2916	Other_MGE	chaperone	TRUE
**Day7**	Colonisation	ANT(4')-Ia	k95_2032	IGE	replication,inversion	TRUE
**Day7**	Colonisation	PC1 beta-lactamase (blaZ)	k95_2137	Phage	infection,regulation	TRUE
**Day7**	Colonisation	PC1 beta-lactamase (blaZ)	k95_2137	Phage	infection,regulation	TRUE
**Day10**	Decolonisation	PC1 beta-lactamase (blaZ)	k95_6598	Other_MGE	chaperone	TRUE
**Day10**	Decolonisation	mecA	k95_2265	Phage	infection,regulation	TRUE
**Day10**	Decolonisation	mecA	k95_2265	Phage	infection,regulation	TRUE
**Day10**	Decolonisation	mgrA	k95_1203	Phage	infection,regulation	TRUE
**Day10**	Decolonisation	lnuC	k95_1310	Phage	infection,regulation	TRUE

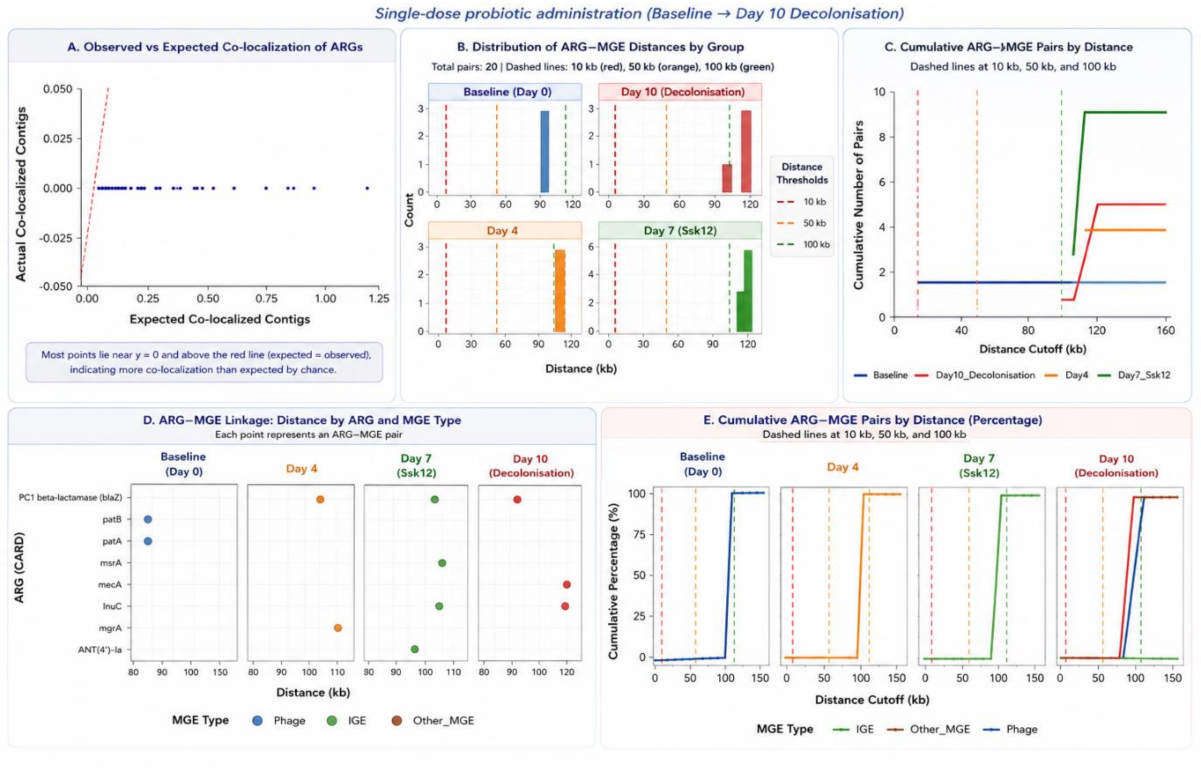

Figure S1. Limited ARG–MGE co-localisation and absence of enriched mobilisable resistance determinants during *Streptococcus salivarius* K12 colonisation.

(A) Observed versus expected co-localisation frequencies of antibiotic resistance genes (ARGs) and mobile genetic elements (MGEs) across all study conditions. Each point represents an ARG–MGE pair identified on the same contig. The dashed diagonal line indicates the expected relationship under the null model. Most observations clustered near the baseline with minimal deviation from expectation, indicating no significant enrichment of ARG–MGE co-localisation beyond stochastic occurrence.

(B) Distribution of ARG–MGE genomic distances across experimental groups, including Baseline (Day 0), Day 4, Day 7 during *S. salivarius* K12 colonisation, and Day 10 following probiotic decolonisation. Histograms represent the number of ARG–MGE pairs detected at increasing genomic distances. Dashed vertical lines indicate conservative proximity thresholds of 10 kb, 50 kb, and 100 kb commonly used to infer putative mobilisation potential. No ARG–MGE pairs were identified within the ≤10 kb threshold at any time point. Most co-localisation events occurred at distances exceeding 90–100 kb, suggesting limited direct mobilisation potential.

(C) Cumulative number of ARG–MGE pairs detected across increasing genomic distance cut-offs. Baseline samples exhibited minimal co-localisation events, whereas

transient increases in cumulative ARG–MGE associations were observed during Day 7 probiotic colonisation and Day 10 decolonisation. However, these events remained restricted to large genomic distances substantially above conservative mobilisation thresholds.

(D) ARG–MGE linkage patterns stratified by ARG identity and MGE category. Each point represents a detected ARG–MGE pair coloured according to MGE type, including phage-associated elements, integrative genetic elements (IGEs), and other mobility-associated elements. ARG-associated MGEs were sporadically distributed across time points, with no evidence of recurrent clustering near high-probability mobilisation distances.

(E) Cumulative percentage distribution of ARG–MGE pairs across increasing genomic distance thresholds. The majority of ARG–MGE associations accumulated only beyond ~90–120 kb, further supporting the absence of high-confidence mobilisable ARGs within the salivary polymicrobial biofilm model. Although transient increases in IGE-associated co-localisation were observed during probiotic colonisation, these remained outside conservative proximity criteria indicative of direct horizontal transfer potential.

Collectively, these findings indicate that transcriptionally active resistome restructuring during *S. salivarius* K12 colonisation was not accompanied by increased high-confidence ARG mobilisation or enrichment of closely linked ARG–MGE pairs under conservative genomic proximity criteria (≤10 kb).

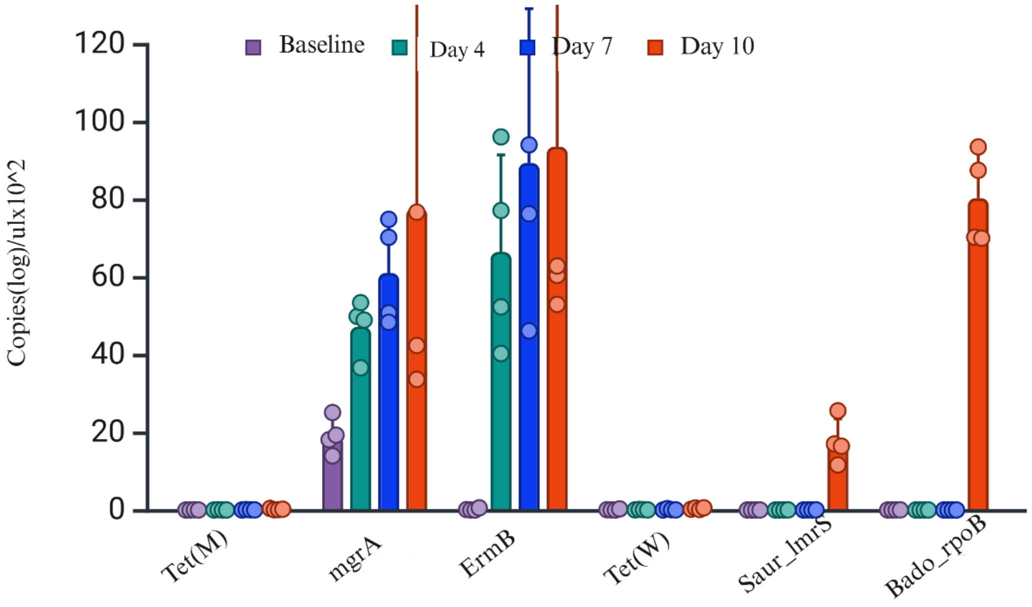

Figure S2. RT–qPCR validation of ARG transcription during *SsK12* colonisation. RT–qPCR quantification of selected ARG transcripts (*tet(M)*, *mgrA*, *ermB*, *tet(W)*, *Saur_lmrS* and *Bado_rpoB*) across baseline and probiotic intervention timepoints (Baseline, Day 4, Day 7 and Day 10). Expression levels are shown as log-transformed copy numbers (log copies $\mu l^{-1} \times 10^2$). Core ARGs (*mgrA* and *ermB*) increased during colonisation, whereas *tet(M)* and *tet(W)* remained low. *Saur_lmrS* and *Bado_rpoB* showed higher expression after decolonisation. Data represent biological replicates with mean values indicated by bars.

References :

1) Roberts MC, Chung WO, Roe D, Xia M, Marquez C, Borthagaray G, Whittington WL, Holmes KK. Erythromycin-resistant Neisseria gonorrhoeae and oral commensal Neisseria spp. carry known rRNA methylase genes. Antimicrob Agents Chemother. 1999 Jun;43(6):1367-72. doi: 10.1128/AAC.43.6.1367. PMID: 10348754; PMCID: PMC89280.
2) Ng LK, Martin I, Alfa M, Mulvey M. Multiplex PCR for the detection of tetracycline resistant genes. Mol Cell Probes. 2001 Aug;15(4):209-15. doi: 10.1006/mcpr.2001.0363. PMID: 11513555.
3) Santos Costa S, Viveiros M, Rosato AE, Melo-Cristino J, Couto I. Impact of efflux in the development of multidrug resistance phenotypes in Staphylococcus aureus. BMC Microbiol. 2015 Oct 24;15:232. doi: 10.1186/s12866-015-0572-8. PMID: 26498754; PMCID: PMC4619429.
4) Mohammed, Rana & Jead, Marwa. (2020). Detection of lmrS, mepA, norC genes among Staphylococcus Aureus Isolates. International Journal of Pharmaceutical Research. 12. 10.31838/ijpr/2020.12.01.243.
5) Lokesh D, Parkesh R, Kammara R. Bifidobacterium adolescentis is intrinsically resistant to antitubercular drugs. Sci Rep. 2018 Aug 9;8(1):11897. doi: 10.1038/s41598-018-30429-2. PMID: 30093677; PMCID: PMC6085307.
